# Sodium Amide Eye Injury: Eye Injury After Sodium Amide Explosion

**DOI:** 10.1155/crop/5017027

**Published:** 2026-09-21

**Authors:** Gretchen Weiss, Cody Manzanero, Cameron McGlone, Brian R. Quaranto, Ruth Marie Mattern

**Affiliations:** ^1^ Jacobs School of Medicine and Biomedical Sciences, State University of New York at Buffalo, Buffalo, New York, USA, buffalo.edu; ^2^ Department of Ophthalmology, Storm Eye Institute, Medical University of South Carolina, Charleston, South Carolina, USA, musc.edu; ^3^ Department of Surgery, Jacobs School of Medicine and Biomedical Sciences, University at Buffalo, Buffalo, New York, USA, buffalo.edu; ^4^ Department of Ophthalmology, Ross Eye Institute, Jacobs School of Medicine and Biomedical Sciences, University at Buffalo, Buffalo, New York, USA, buffalo.edu

**Keywords:** alkali corneal burn, case report, chemical eye injury, sodium amide

## Abstract

Alkaline chemical injuries are a common ocular trauma, with potentially devastating consequences. We report the initial 36‐month course of healing of a 23‐year‐old male with bilateral partial chemical burns of the eyes from an industrial sodium amide explosion. To our knowledge, this is the first specific report of a case of ocular chemical injury with sodium amide. Persistent epithelial defects of cornea and conjunctiva, tissue melting, fibrovascular pannus, and symblepharon were consequences of this injury. Medical management, amniotic membrane application, and resection of fibrovascular tissue achieved limited control of the sequelae of the injury.

## 1. Introduction

Sodium amide, also called “sodium azanide” or “sodamide,” is a strong base used in industrial synthesis of organic compounds [1]. In general, alkali injuries of the eyes can result in corneal opacification and vascularization, scleral or corneal melts, persistent epithelial defects, fibrovascular pannus, and symblepharon formation. These problems occurred in the case of sodium amide eye injury that we report here.

## 2. Case Presentation

**Figure 1:**
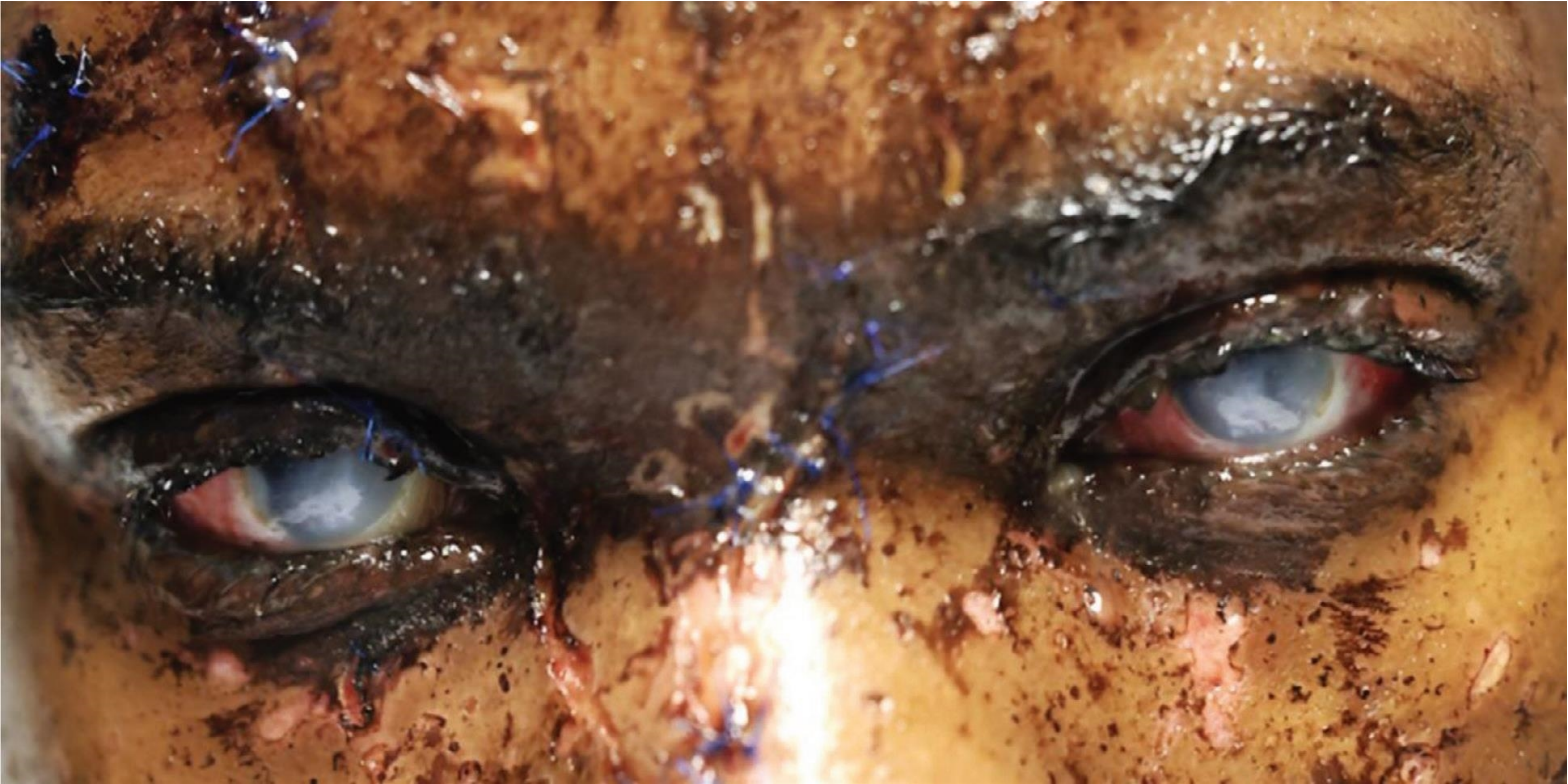
Eye and facial injury after sodium amide explosion.

A 23‐year‐old male with no prior ocular or medical history was cleaning a glass storage container of sodium amide in his work at an industrial plant. An explosion of the sodium amide sent the chemical and shards of glass into his eyes, face, and neck. Since he had immediate blurred vision and pain, he irrigated his eyes with tap water for 15 min on site before going to the emergency department. There, the eyes were irrigated with seven liters of sterile water and normal saline through Morgan lenses. The fornices were swept to remove any residual glass fragments or residual chemical material. Dense opacification was present in the lower 2/3rds of both corneas, and extensive limbal blanching was present in both eyes. Skin lacerations were sutured (Figure 1).

Medications prescribed, as recommended in the literature [2–5], included prednisolone 1% (4 times per day as anti‐inflammatory), moxifloxacin (4 times per day as infection prophylaxis), vitamin C 1500 mg orally (daily to promote corneal collagen healing), doxycycline 50 mg orally (twice per day to reduce inflammation from matrix metalloproteinases), and preservative‐free artificial tears (every 2 h to promote epithelial healing). Doxycycline and steroids are known to reduce inflammation [6], and inflammation control is important in management of chemical burns [7]. Oral ascorbate (vitamin C) restores aqueous levels of ascorbic acid, reduces collagen degradation, and reduces risk of corneal ulceration and perforation [2].

At his first ophthalmology outpatient visit on Day 6 postinjury, Snellen uncorrected visual acuity at distance was 20/300 right eye and 20/200 left eye. Intraocular pressures in both eyes were normal and remained normal on most succeeding visits; however, brimonidine 0.2% was prescribed as glaucoma prophylaxis. Limbal ischemia and corneal epithelial defects were present bilaterally except for two clock hours in the right eye and three clock hours in the left eye. Dense corneal opacification was present bilaterally. The conjunctiva was diffusely injected in both eyes, but no areas of conjunctival necrosis were noted.

Amniotic membranes of 12.0 mm diameter (BioD Optix, Integra) were placed on both corneas under bandage contact lenses. (The use of amniotic membranes for chemical burns is discussed below). Prednisolone was tapered off within a week following the “general consensus” guidelines in the literature [3] to reduce risk of corneal melts, but the other medications were continued.

The patient′s course is summarized by problems.

### 2.1. Pain and Photophobia

The patient had bilateral photophobia and pain during the first 6.5 weeks after the injury.

Gabapentin 300 mg three times per day was prescribed and cooled artificial tears were used. Topical atropine 1% twice per day, an antagonist to muscarinic cholinergic receptors, was also used (following common practice) to reduce ciliary spasm and pain [8].

The pain and photophobia gradually resolved; an occasional dull ache remained for 12 months.

### 2.2. Tissue Melting

**Figure 2:**
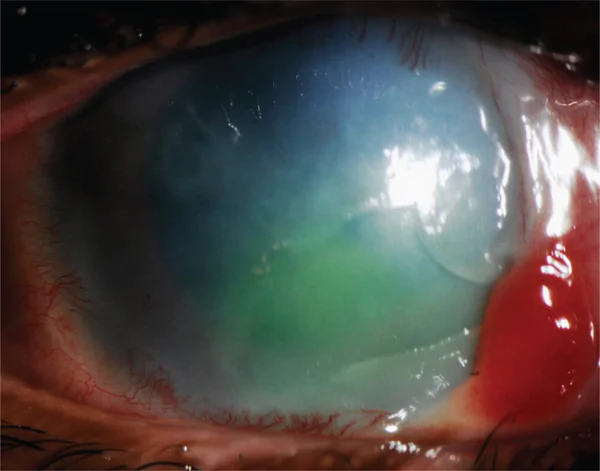
Right eye, 6 weeks postinjury. A pyogenic granuloma is present over the area where conjunctiva and sclera had melted. A persistent epithelial defect overlies 37% of the cornea.

Three weeks after the injury, the right eye developed one clock hour of scleral thinning and a gap in the conjunctiva at the 4 o′clock position. The scleral thinning worsened to 50%, but then improved. A pyogenic granuloma formed overlying this location at 5 weeks postinjury (Figure 2) and gradually resolved by 2.5 months.

In the left eye, a gap formed in the conjunctiva from 4 to 6: 30 o′clock; however, no scleral thinning occurred, and the overlying tissue sealed. Corneal thinning in a nasal area of the left cornea occurred but was not visually significant since it was peripheral and epithelium filled in.

### 2.3. Corneal Epithelial Defects

In the left eye, the epithelium over the cornea was 60% sealed by 2.5 weeks postinjury, 85% intact by 7.5 weeks, and 100% complete by 2 months. The right eye corneal epithelium was slower to heal; it took 3.25 months for the epithelial defect to decrease to 1 mm in diameter. The patient was then temporarily lost to follow‐up due to insurance issues, but the epithelium was sealed when he was examined at 4.75 months postinjury.

### 2.4. Fibrovascular Pannus

In both eyes, fibrovascular pannus invading the cornea started at 2.75 months. Such pannus has been noted in the literature to occur in some cases of severe ocular injury [3, 9].

Our working hypothesis was that inflammation contributed to fibrovascular pannus. Some studies in the literature indicate that this hypothesis is a reasonable one. Histology studies of fibrovascular pannus in alkali cornea burns have shown many inflammatory cells [10, 11]. Kethiri et al. also reported histological and immunohistochemical studies of pannus in alkali burn cases, finding evidence of considerable immune cell presence and especially T‐cell infiltration [12].

Thinking that significant inflammation played an important role in the fibrovascular pannus in our case, we prescribed multiple anti‐inflammatory medications. Since the epithelium was nearly sealed at this point, the corneal melting effect of topical steroid was no longer a significant risk [3, 4]; prednisolone 1% was restarted at three times per day and increased by steps to eight times per day. Doxycycline was continued due to its anti‐inflammatory effect on metalloproteinases. Ointment containing dexamethasone was prescribed twice a day to provide further topical steroid anti‐inflammatory treatment. Tacrolimus 0.03% ointment was also ordered. Tacrolimus is anti‐inflammatory as a calcineurin inhibitor of T cell activity; it decreases formation of interleukin‐2 as well as other cytokines [13, 14]. However, it was not immediately available to this patient due to insurance issues. Topical cyclosporine, another T‐cell inhibitor, was prescribed for use twice per day. Cyclosporine as well as tacrolimus, steroids, and oral tetracyclines are well known as anti‐inflammatory agents to help restore a damaged ocular surface and reduce epitheliopathy [7]. Preservative‐free artificial tears were also continued to promote epithelial healing.

**Figure 3:**
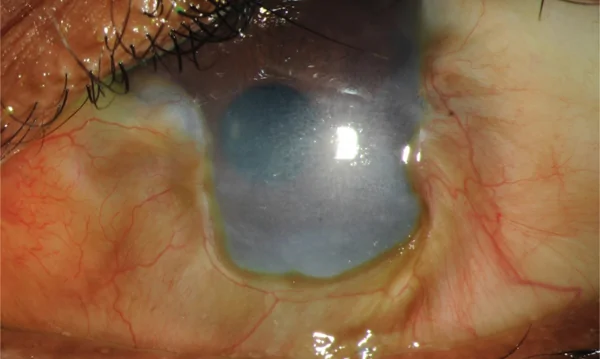
Left eye, 6.25 months postinjury. Fibrovascular pannus covers portions of the nasal, inferior, and temporal cornea.

Fibrovascular pannus continued to grow. In the better‐seeing left eye, growth of this tissue threatened the pupillary zone (Figure 3).

Our clinical decision‐making at this time was affected by the significant risks and contra‐indications to other possible treatments for ocular chemical injury. Keratoprosthesis is one approach; but the risks of endophthalmitis, retinal detachment, device extrusion, and glaucoma [15–17] make this option more appropriate for cases with very severe damage. Limbal stem cell autografts were not feasible here due to the bilateral nature of this injury, and limbal allografts from living relatives or cadavers would require long‐term strong systemic immunosuppression and have substantial risks of complication and failure [18, 19]. Cultured limbal stem cell autograft was not an option at our institution due to the lack of a qualified laboratory to perform such a clinical procedure. In addition, such surgical intervention would need to wait until inflammation was controlled [3, 7]. Another option for treatment might be use of antivascular medications. Though antivascular agents have been studied for their role in treating corneal vascularization [20–22], there is no established protocol for treating fibrovascular pannus in chemical burns with such medications.

The literature refers to some cases in which peeling (also called “resection” or “debridement”) of fibrovascular pannus has been performed, sometimes combined with application of amniotic membrane after the resection or as a prelude to other procedures [3, 23–25]. In our case, we performed peeling of the nasal and superonasal fibrovascular tissue in the left eye at 6.5 months postinjury. An amniotic membrane (Xcellerate amniotic membrane, Precise Bioscience) was applied to the area after peeling and held in place by tissue glue (TissueSeal histoacryl adhesive, Fuller Co.) and a bandage lens. Topical triamcinolone (5 mg) was also applied. Resection of dense pannus was indicated at this time to protect the pupillary zone from migrating tissue, which threatened vision in the better‐seeing eye.

Incision into inflamed tissue incites aggressive regrowth. After the debridement, fibrovascular pannus recurred. At nearly 7 months postinjury, a larger resection of invading fibrovascular tissue in the left eye was performed with undermining of the conjunctiva beyond the limbus from 8 to 11 o′clock, placement of another amniotic membrane (BioD Optix, Integra) secured by a nylon 10‐0 suture and TissueSeal glue, and application of triamcinolone (5 mg) to quiet inflammation. After this procedure, the medications were continued as described earlier.

Fibrovascular pannus did not recur in the superonasal quadrant of the left cornea, now with 29 months of follow‐up.

**Figure 4:**
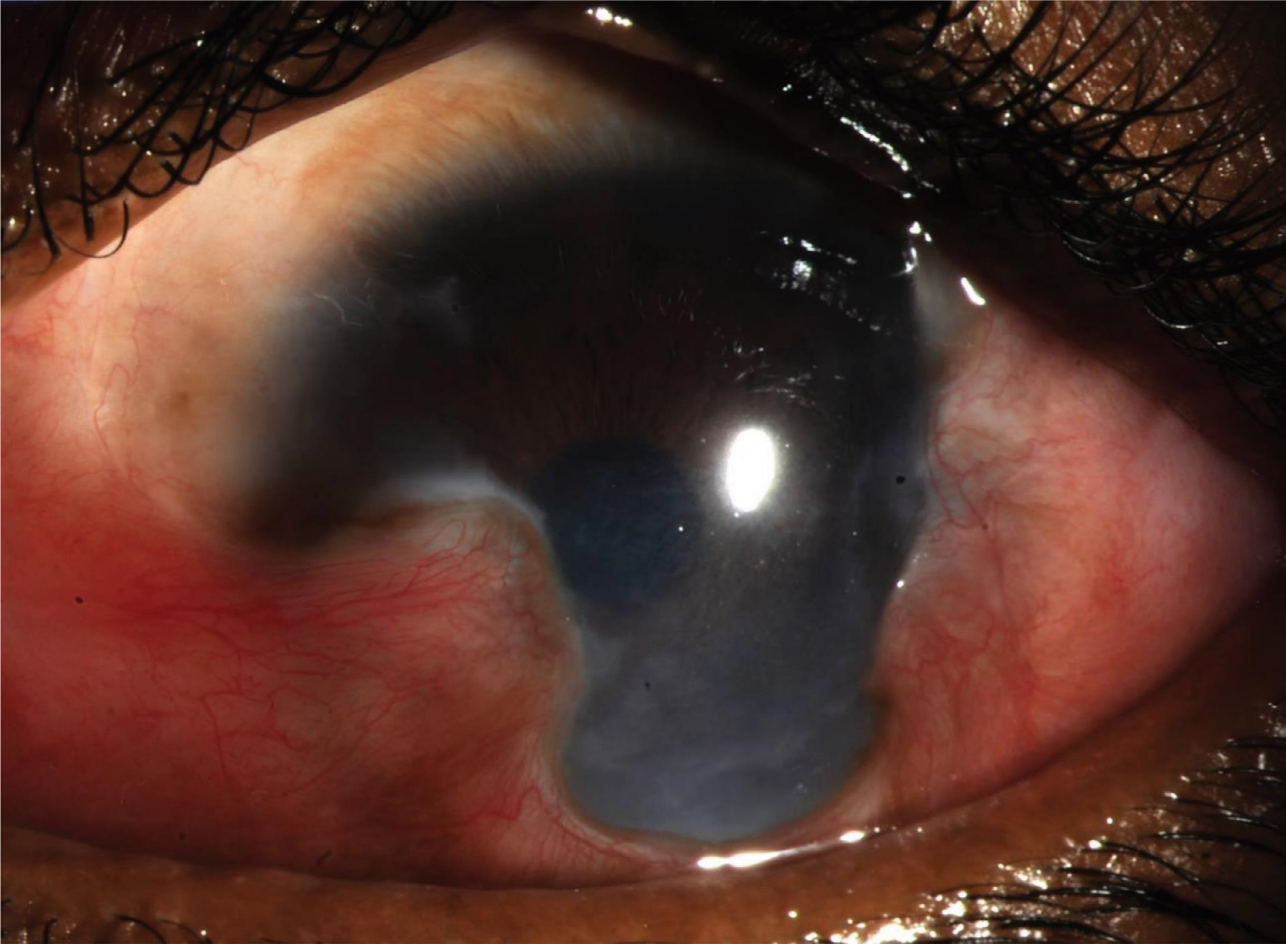
Left eye, 1 year postinjury. Fibrovascular pannus on the cornea grows in from the inferonasal aspect of the eye. The temporal and inferior limbus is also covered with fibrovascular pannus.

Fibrovascular pannus for the right eye was deferred at this point; since the pannus was more peripheral in this eye, arresting it was less urgent. The right eye was maintained on anti‐inflammatory treatment matching the medical regimen of the left. However, fibrovascular pannus in the right eye gradually increased. Meanwhile, in the left eye fibrovascular pannus gradually progressed in the inferonasal quadrant in the months after the peeling procedures in the seventh month (Figure 4).

Peeling the fibrovascular tissue off the cornea, injection of triamcinolone 5 mg, suturing amniotic membrane (BioD Optix), and placement of bandage contact lenses were performed on both eyes at 13.5 months postinjury. The debridement was performed only for the areas that had shown recent active growth, leaving stable fibrovascular tissue untreated to limit postprocedure inflammation. The amniotic membrane in the right eye was dislodged and lost within hours after the procedure. In the left eye, the amniotic membrane and bandage lens remained in place until the membrane dissolved.

After the procedure the previous anti‐inflammatory medications were continued, including topical prednisolone in both eyes at 4 times per day; tacrolimus ointment 0.03% was then available for the patient at three times per day after removal of the bandage lens. In addition, oral prednisone 80 mg daily was prescribed for better inflammation control and tapered slowly over 6 months.

**Figure 5:**
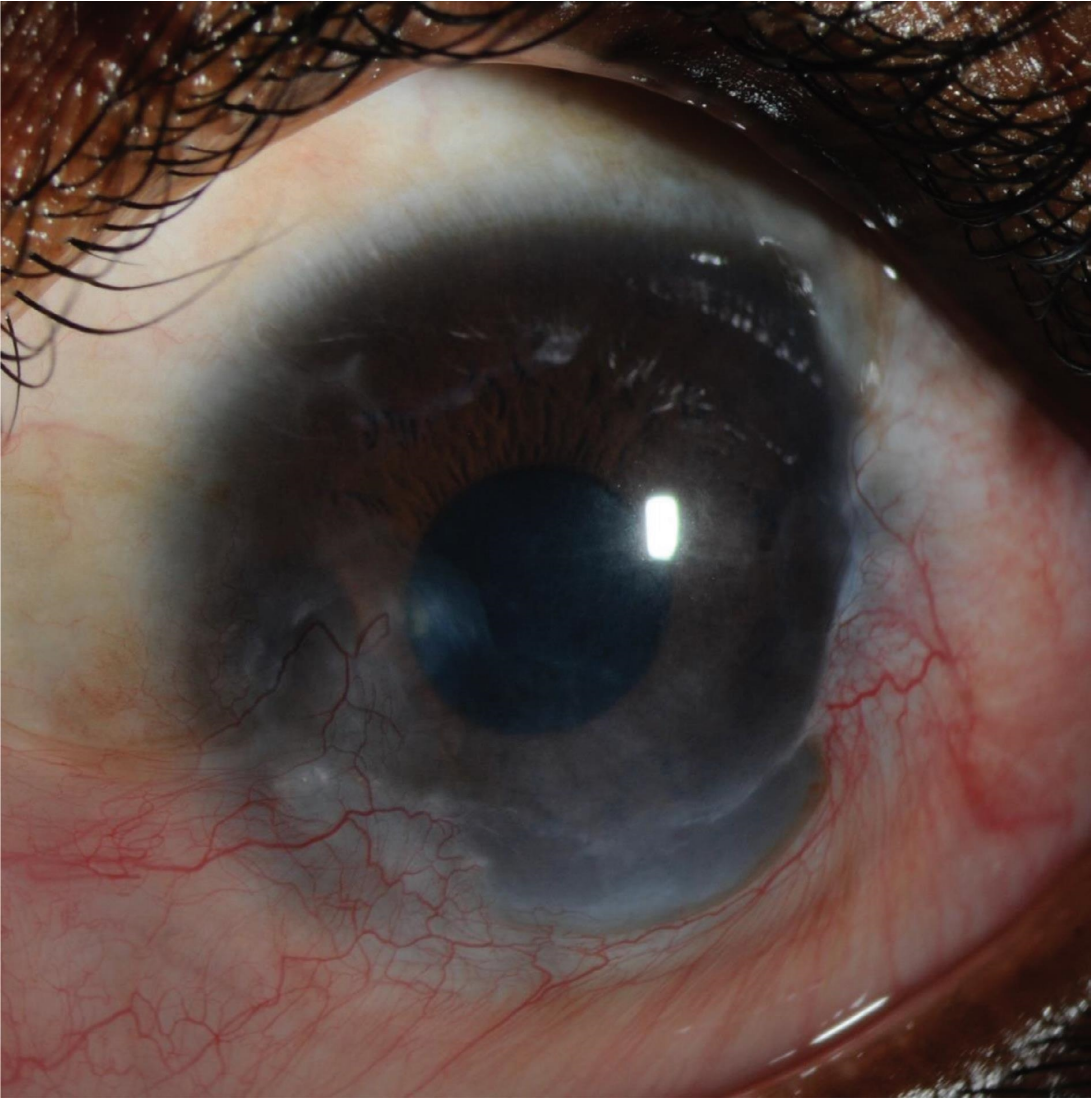
Left eye, 3 years postinjury and 22.5 months after peeling of fibrovascular pannus inferonasally. The superonasal limbus remains free of pannus with 29 months of follow‐up after pannus resection twice in the seventh month. The corrected visual acuity of the eye is 20/25 with pinhole improvement to 20/20.

**Figure 6:**
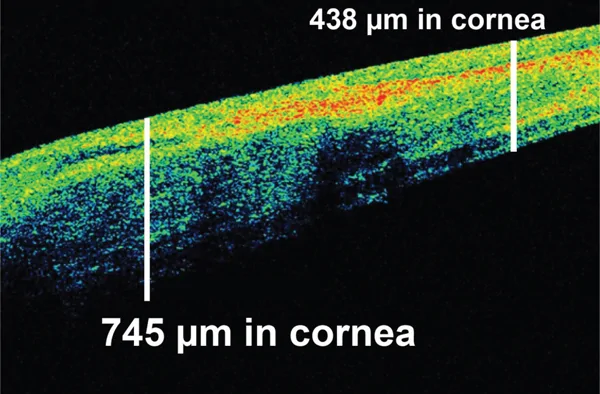
Left eye, 3 years postinjury. Anterior segment ocular coherence tomography image of inferonasal left eye showing recurrent pannus overlying the inferonasal peripheral cornea, with clear cornea on the right side of the image.

Two months after the procedure, at 15.25 months postinjury, new fibrovascular invasion started in the left eye inferonasally. This pannus increased gradually. At 36 months postinjury, the pannus remains (Figure 5), without further inferonasal corneal ingress in the past 16 months. The anterior segment ocular coherence tomography image (Figure 6) shows the thickening of tissue with continued presence of pannus in the left eye at 3 years postinjury.

**Figure 7:**
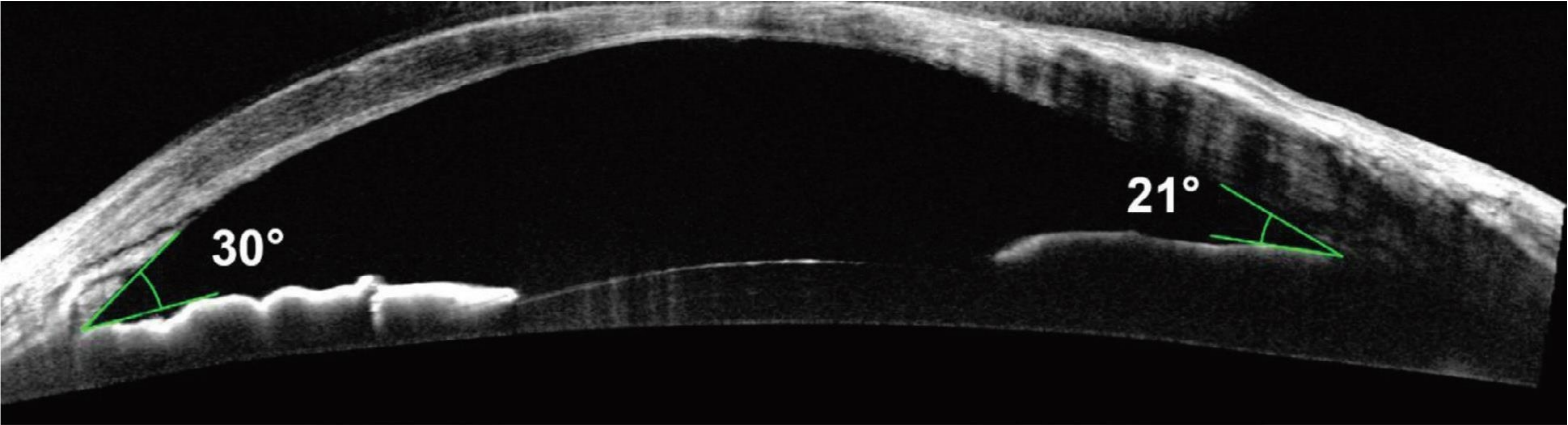
Right eye, 3 years postinjury. Anterior segment ocular coherence tomography horizontal central image. The temporal angle measures 30°, whereas the nasal angle measures only 21°.

The right eye at 3 years postinjury has more damage nasally than temporally, as is already suggested by the persistent inflammation in the nasal aspect of that eye compared with the relatively quiet temporal aspect. Anterior segment ocular coherence tomography shows the cornea with fibrovascular pannus in the nasal aspect of the right eye (Figure 7). Measuring over 1 mm, the cornea with recurrent pannus is significantly thick compared with a normal peripheral corneal thickness of 610–670 *μ*m.

It is well known that alkaline chemicals can cause intraocular damage because of their ability to penetrate tissues [3]. Testing is consistent with mild anterior segment intraocular damage to the nasal aspect of the right eye. The anterior segment ocular coherence tomography shows a nasal chamber angle measuring only 21° nasally, which is below normal (Figure 7). In contrast, the temporal anterior chamber angle measures 30°, falling within the range of normal Grade 3 open angles (25°–35°). (Figure 7).

### 2.5. Symblepharon

**Figure 8:**
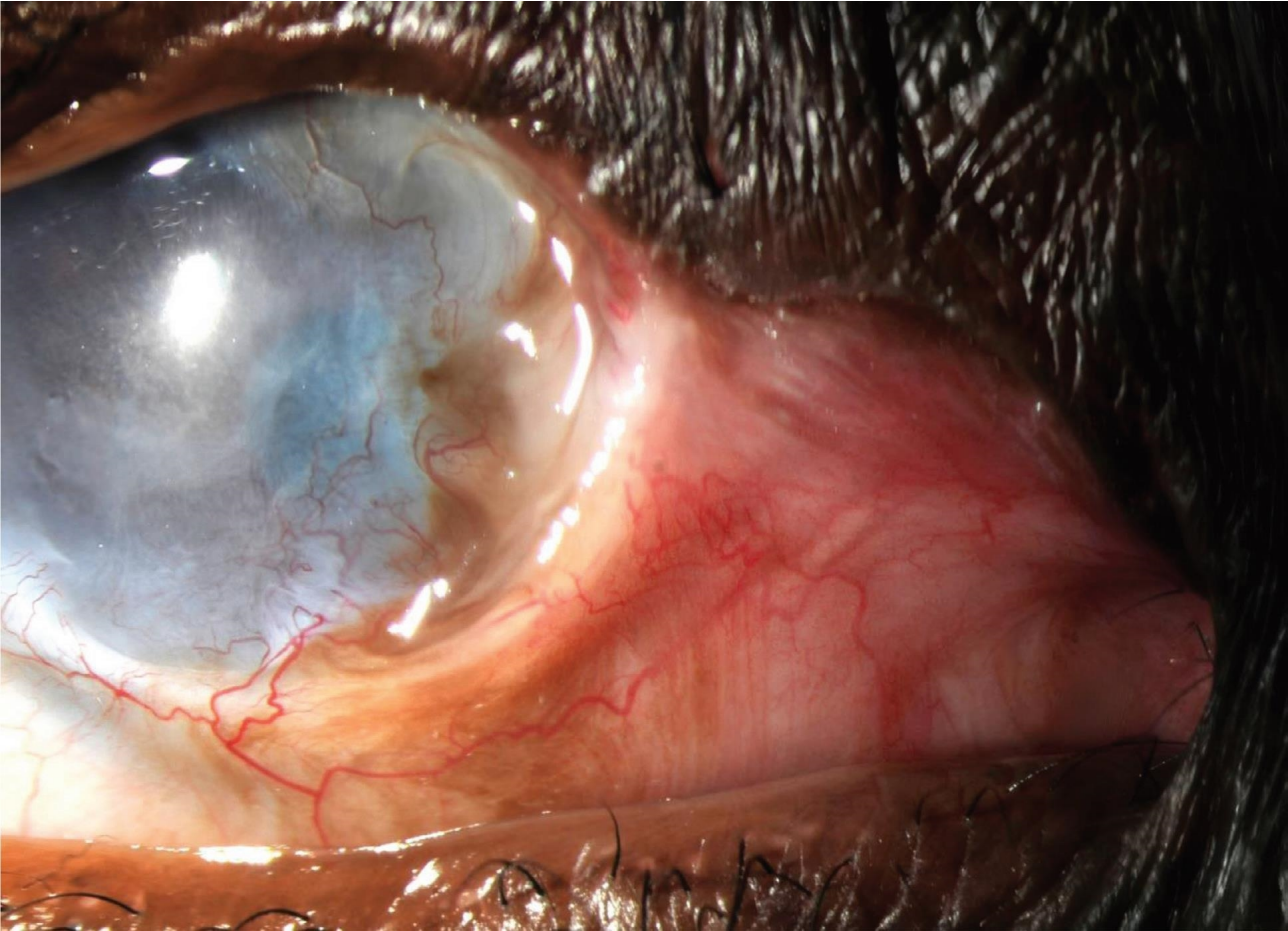
Right eye, 20 months postinjury. The recurrent symblepharon at the nasal aspect of the eye joins the upper lid to the globe for 10 mm.

A symblepharon of the nasal aspect of the right eye and upper lid was removed by a plastic surgeon at 10.25 months postinjury. However, this symblepharon recurred. At 20 months postinjury (Figure 8), the symblepharon extended inferonasally with 10 mm of attachment between globe and upper lid. There was also a small lower lid symblepharon.

Repeat symblepharon removal in the right eye was performed at 34 months postinjury. The inferior symblepharon has remained lysed, but the upper one has recurred at 2 months postoperatively in a strand with width of 2 mm. Further removal of the symblepharon could be done after allowing more time for quieting of inflammation.

### 2.6. Patient Compliance and Intervention Tolerability

Throughout the postinjury course, the patient′s eyes have been monitored with frequent follow‐up visits. The patient has not missed any visit, except when insurance issues caused cancellation of his appointments. Frequent visits allowed close monitoring of the eyes, encouraged compliance, and facilitated timely adjustments and updates of medication refills.

The patient′s injury resulted in a phobia about instruments coming toward his eyes. Procedures performed in the clinic were difficult for the patient, so subsequent procedures were performed in the operating room with intravenous sedation or general anesthesia.

### 2.7. Vision

The left eye had uncorrected distance acuity of 20/200 postinjury on the first outpatient visit. Usual uncorrected acuities in that eye improved to 20/70 by 2 months, and to 20/50 by 3 months. In the second 6‐month period of the first year after the injury, visual acuity was 20/25 or 20/30 on 57% of the visits, and in the 20/40 to 20/60 range on 43% of the visits.

In the last half of the third year postinjury (Months 30 through 36), the left eye had no visits with uncorrected acuity less than 20/40. A total of 64% of visits recorded uncorrected acuity of 20/25 or 20/30, and 91% had pinhole acuity of 20/20 or 20/25. Refraction at 36 months yielded 20/25 vision.

The right eye, which had 20/300 uncorrected distance acuity on the first outpatient visit after the injury, continued to have worse vision than the left eye throughout the postinjury period. Uncorrected acuity during the second half of the first year was 20/200 or 20/250 on 85% of visits and 20/300 or 20/400 on 10% of visits, a significant difference from the left eye in which 100% of acuities during that period were 20/60 or better.

In the most recent 6‐month period of the third year postinjury (Months 30–36), for the right eye, no uncorrected distance acuity was better than 20/100, and none was worse than 20/300. Acuity was 20/300 on 11% of visits, 20/100 on 22% of visits, and 20/150 to 20/250 on 73% of visits. Pinhole improvement of acuity was 20/80 or 20/100 on 91% of the visits in which uncorrected acuity was less than 20/100. However, no pinhole acuity was better than 20/80 for the right eye.

### 2.8. Method of Literature Search

We searched the medical literature for other reports of eye injury from sodium amide. PubMed was searched for references to sodium amide eye injury by using Boolean operators AND and OR in the following search input: {“sodium amide” OR “sodamide” or “sodium azanide”} AND (“eye injury” OR “corneal burn” OR “eye trauma” OR “ocular injury” OR “ocular trauma”). Zero results in PubMed for this search were found. The search was performed without date restrictions, and all items in the database were searched including title, abstract, keywords, and text.

The same method was used to search the EMBASE database, with maximum scope of search and without date restrictions on the articles searched. This search also had negative results.

The searches used English terms as search phrases. This is a limitation of the searches, since it is possible that there were articles on sodium amide eye injury that could not be found with such search terms.

We also searched comprehensive databases in the chemistry literature. The Haz‐Map database on Hazardous Chemicals and Occupational Disease [26] and the CAMEO Chemicals Database of Hazardous Chemicals [27] state that sodium amide can cause “caustic burns of eyes and skin.” However, no specific cases or general summaries are cited, and a link to PubMed in the Haz‐Map database gives zero results on eye injuries by sodium amide.

The Hazardous Substances Data Bank [28], a “toxicology database that focuses on the toxicology of potentially hazardous chemicals,” lists 134 articles in a search on sodium amide. However, perusal of all of these articles shows nothing relevant to eye injury caused by sodium amide.

## 3. Discussion

### 3.1. Comparison With Previously Reported Chemical Injuries

How does this case of sodium amide injury compare with other cases of ocular chemical injuries?

First, how does this case measure up to expectations for postinjury course compared with other cases in the literature?

Two of the chemical injury grading systems, which outline expectations for postinjury course based on initial presentation, provide a framework for comparison of our case with other cases of chemical injuries.

By the Roper‐Hall classification system of chemical burns [29], written in 1965 but still widely used, our case would be classified as a Grade IV injury, since there was greater than 50% limbal ischemia in both eyes. Such an injury, falling within the most serious Roper‐Hall grade, carries a “poor” prognosis.

The classification scheme of Dua et al. [30] notes the wide variety of severity of injury within Roper‐Hall Grade IV, and divides advanced chemical injuries into three categories, IV, V, and VI. Based on extent of initial limbal involvement, the right eye in our case at initial presentation would be labeled a Dua Grade V chemical injury with “guarded to poor” prognosis, the left eye a Grade IV injury with “good to guarded” prognosis.

How does our case compare so far with outcomes, judged in relation to peer classes of injured eyes on the Roper‐Hall and Dua grading systems of initial postinjury examinations?

The left eye has achieved functional vision of 20/50 by 3 months postinjury, and since then has frequently been 20/25 or better. In the superonasal area of our patient′s left eye, peeling the fibrovascular tissue in the seventh month was followed by persistent absence of fibrovascular pannus during 29 months of follow‐up. This limited success brought the total length of limbus without such pannus to five clock hours.

The right eye has been less successful than the left eye so far, with only modest vision gain of 1–3 lines on the Snellen acuity chart. Nevertheless, the right eye has so far avoided conditions associated with poor prognosis, such as progressive deterioration of vision, severe corneal melting, perforation, or loss of the eye. In months 30 through 36 postinjury, the right eye on 91% of the visits showed better acuity than on the initial postinjury Snellen chart measurement of acuity.

Second, how does this case of sodium amide injury compare with expectations in the literature about speed of epithelial healing in chemical injuries?

Some say that the prognosis is poor in corneal chemical injury if the epithelium is not healed within 3 weeks [31], and “failure to epithelialize within 21 days” is sometimes used as an outcome measure in analyses of healing [32]. However, these are statements of expected timeline of epithelial healing for eyes with all grades of chemical injury, not just severe burns. Expectations projected by timelines of typical healing in ocular burns need to be tempered for severe chemical injuries. In the present case, epithelial healing was slow, requiring 2 months for the left eye and over 3 months for the right eye. The left eye in our case healed faster than the average for Roper‐Hall Grade IV burns stated in one clinical trial in the literature (60 days compared with 75.8 days) [33]. The right eye time to epithelialization was greater than this report of average epithelial healing time of 75.8 days, but within the range documented in that trial (46–170 days) [33]. (As noted previously, we do not know the exact time of closure of epithelial gaps in the right eye in our case, since the patient was missing from clinic for weeks due to insurance issues. However, we do know that the epithelial healing time was between 90 and 143 days).

Third, how does this case of fibrovascular pannus compare with previous cases of pannus post chemical injury?

There are prior mentions in the literature of fibrovascular pannus occurring in chemical injury to the eyes, and of treatment including pannus resection as a step followed by amniotic membrane procedures or limbal stem cell transplantation [3, 34]. These reports show generally good results, but with some cases of recurrence of pannus [34]. Our case, which did involve pannus recurrence, is not unique. However, the literature on fibrovascular pannus is not large enough to generate consensus generalizations about the risk of recurrence and the factors controlling good outcomes.

### 3.2. Role of Amniotic Membrane in Treatment

Now widely used in ophthalmology, the purported ability of amniotic membranes to protect the cornea, quiet inflammation, prevent central corneal vascularization, treat corneal ulceration, reduce pain, prevent fibrosis and promote epithelial recovery is touted by various advocates of their use [7, 34–44]. Others, however, question the soundness of the grounds for using amniotic membranes for such treatment [32, 33]. For example, one clinical trial notes a longer average time to epithelialization with the use of amniotic membrane than without [33]. In addition, a meta‐analysis of randomized controlled trials on the use of amniotic membrane after chemical injuries pointed to the uncertainty of evidence in these trials [32].

One problem in setting up high‐quality controlled trials is the difficulty in standardizing the cases and controls, and the variability of donor and host clinical situations. For example, the clinical trial mentioned above used cases of Grade IV Roper‐Hall chemical injuries [33], but as Dua pointed out, there is wide variety in severity of injury within that grade [30]. In addition, standardization for controlled trials is challenging due to the variety of types of amniotic membranes—cryopreserved, dehydrated, lyophilized, and decellularized tri‐layer basement membrane—now available through multiple different commercial outlets with multiple different processing techniques. Finally, some of the purported ways in which amniotic membranes are thought to be helpful, such as pain mitigation and reduction of inflammation, are difficult to measure precisely and so are not easily amenable to use as good outcome parameters in clinical trials.

In addition to questions about proof of the efficacy of amniotic membranes, a handful of reports about deleterious effects of such membranes, specifically microbial or fungal keratitis, should be noted [45–47]. However, such infectious keratitis in amniotic membrane cases is rare [48], and it is therefore difficult to assess the frequency of adverse events in clinical trials [33].

Though there is insufficient high‐quality evidence to support the use of amniotic membranes, use of this modality as “supportive rather than definitive” treatment in chemical injuries may be warranted [44]. Clinicians need to do the best they can to help patients with severe eye problems, and treatment cannot be delayed waiting for high‐level evidence [4, 43]. For example, the consensus guidelines on limbal stem cell deficiency endorse amniotic membrane use even while acknowledging the lack of high‐level evidence for its efficacy and calling for more research in this area [7].

### 3.3. Future Directions

For the right eye, additional resection of fibrovascular pannus may be warranted. For the stromal opacification, corneal transplantation might be an option in the future, but before adequate restoration of a functional ocular surface, it would be doomed to failure [7, 9]. Cultured limbal stem cell transplantation may be an option when facilities for this procedure become available to the patient and would pose less risk to his left eye than a simple transplant of limbal stem cells from the left eye to the right.

### 3.4. Limitations of This Report

This report is significantly limited by the fact that it discusses only a single case. We do not know whether sodium amide is in general prone to cause such problems as occurred in the postinjury course of our patient. In addition, because this is a single case in which multiple methods were employed, we cannot determine with accuracy the role of each of them in contributing to the success or failure of the interventions.

Though the tissue growing onto the cornea after the injury had the clinical appearance of fibrovascular tissue, similar to the fibrovascular pannus in chemical injuries discussed in the literature, we lack histopathological evidence and immunohistochemical analysis to prove this clinical impression.

An additional limitation is the absence of quantitative assessments of degree of corneal opacification, vascularization, and inflammation during the postinjury healing. Our quantitative measurements did include measures of visual acuity and size of epithelial defects.

Another limitation is the open‐ended future of the healing process in this case. During succeeding years of this young patient′s life, even more problems may be encountered and even more improvements may be realized. Determination of ultimate outcome is still well in the future.

## 4. Conclusion

Explosion of a glass container of sodium amide led to an eye injury with persistent epithelial defects, tissue melting, symblepharon, and invasion of fibrovascular tissue onto the corneas. Literature search using English search terms did not find any prior specific reports of sodium amide eye injuries in the biomedical literature or in the chemical literature, though the chemical literature did acknowledge without elaboration that sodium amide is one agent that can cause caustic burns of eyes and skin.

Both eyes in this case had delayed epithelial healing of the corneas. Fibrovascular pannus occurred in the corneas of both eyes, a complication which has been noted in the literature in some serious chemical injury cases. Mechanical peeling of the fibrovascular tissue encroaching on the cornea was accompanied by limited arrest of this invasion, when combined with medical anti‐inflammatory treatment and application of amniotic membranes. Some fibrovascular pannus did have recurrence after treatment, and retreatment was partly successful in achieving prolonged arrest of recurrence. One eye has achieved excellent vision, whereas the other eye still has significant vision limitations. Both eyes have so far avoided the devastating ocular problems associated with many severe chemical injuries, but at 3 years postinjury the healing is still in progress.

## Author Contributions

All authors contributed to the conception, data acquisition or interpretation, and composition or critical revision of early or later drafts of the manuscript.

## Funding

No funding was received for this manuscript.

## Disclosure

All authors read the final version of the manuscript and accept responsibility for the work. All attest that they meet current ICMJE criteria for authorship.

## Ethics Statement

The patient gave written informed consent for the publication of this report and accompanying images. In accordance with local regulations, institutional review board review is not required for case reports. Artificial intelligence was not used in any aspect of this project or in the manuscript content.

## Conflicts of Interest

The authors declare no conflicts of interest.

## Supporting information


**Supporting Information** Additional supporting information can be found online in the Supporting Information section. File S1: CARE Case Report Guidelines; Timeline; Roper‐Hall classification of severity of ocular surface burns; and Dua, King, and Joseph classification of severity of burns.

## Data Availability

Data generated and analyzed are included in the article. Further questions may be addressed to the corresponding author.
