# Supplementary material for: Sodium Amide Eye Injury: Eye Injury After Sodium Amide Explosion
Source: Case Rep Ophthalmol Med. 2026 Sep 21;2026:5017027. doi: 10.1155/crop/5017027 (PMC13592139; doi:10.1155/crop/5017027)
Supplement: Supplementary file 1 [file CROP-2026-5017027-s001.docx]

SUPPLEMENTARY MATERIAL: SODIUM AMIDE EYE INJURY

CARE Case Report Guidelines

TABLE 1: Timeline

TABLE 2: Roper-Hall Classification of Severity of Ocular Surface Burns

TABLE 3: Dua, King, and Joseph Classification of Severity of Burns

CARE Case Report Guidelines

Title

1. The diagnosis of primary focus, followed by “Case Report”	Title line

[“Case Report” omitted from title as per journal guidelines]

Key Words

2. 2 to 5 key words that identify diagnoses or interventions in this case report

including “case report”	Line 10

Abstract

3a. Introduction: What is unique about this case and what does it add to

the scientific literature? Line 1-9

3b. Main symptoms and/or important clinical findings	Line 6-7, 52-57

3c. The main diagnoses, therapeutic interventions, and outcomes Line7-9

3d. Conclusion—Main “take-away” lesson(s) from this case	Line 7-9

Introduction

4. One or two paragraphs summarizing why this case is unique	Line 1-6, 11-16

Patient Information

5a. De-identified patient-specific information	Line	18-19

5b. Primary concerns and symptoms of the patient	Lines 19-21

5c. Medical, family, psycho-social history, genetic	N/A

5d. Relevant past interventions with outcomes	N/A

Clinical Findings

6. Significant physical examination and important clinical findings --Lines 24-26, 39-45

Timeline

7. Information from this episode of care organized as a timeline--Supp. Table 1

Diagnostic Assessment

8a. Diagnostic testing	N/A

8b. Diagnostic challenges (such as access to testing, financial, cultural) N/A

8c. Diagnosis (including other diagnoses considered)	N/A

8d. Prognosis	Lines	261-306

Therapeutic intervention

9a. Types of therapeutic intervention

Lines 18-205

9b. Administration of therapeutic intervention

Lines 18-205

Follow-up and Outcomes

10a. Clinician and patient-assessed outcomes (if available)	Line 214-234

See patient statement below

10b. Important follow-up diagnostic and other test results	Lines 162-190

10c. Intervention adherence and tolerability	Lines 206-213

10d. Adverse and unanticipated events

Lines	131-2, 204-5

Insurance issues	Lines 75-76, 96

Discussion

11a. A scientific discussion of the strengths AND limitations associated with this case report Lines 236-259,

11b. Discussion of the relevant medical literature with references

Lines 121, 108-120, 236-249, 262-350

11c. The scientific rationale for any conclusions	Lines 262-357

11d. Primary “take-away” lessons of this case report in a one paragraph conclusion

Lines 377-392

Patient Perspective

12. The patient should share their perspective on the treatment(s) they received.

Patient statement:

“I am satisfied with the progress that’s been made, overall. I am pleased with the doctors’ work since the incident.”

Informed Consent

13. Did the patient give informed consent? Yes

TABLE I. Timeline of Major Events

FVP = fibrovascular pannus

**Time Post-injury	Right Eye	Left Eye**

6 days	Amniotic membrane #1	Amniotic membrane #1

3 weeks	Scleral melting 1 clock hour	Conjunctival defect

Conjunctival defect

5 weeks	Pyogenic granuloma	Conjunctiva intact

2 months	Symblepharon nasal	Corneal epithelium intact

2.5 months	Pyogenic granuloma resolved

2.75 months	Epithelial defect 1.25 mm	FVP starts

3.25 months	Epithelial defect 1.0 mm

4.75 months	Corneal epithelium intact

6.5 months	Peeling #1 of FVP

Amniotic membrane #2

6.75 months	Peeling #2 of FVP

Amniotic membrane #3

10.25 months	Symblepharon excised

13.5 months	Peeling #1 of FVP v	Peeling #3 of FVP

Amniotic membrane #2	Amniotic membrane #4

15.25 months	FVP, 1 mm onto cornea

16.25 months	FVP. 2.5 mm onto cornea

20 months	Ridge of elevated scar tissue	FVP, 2.5 mm onto cornea

24 months	Gradual recurrence of FVP

34 months	Symblepharon excision #2

TABLE 2. Roper-Hall Classification of Severity of Ocular Surface Burns [29]

**Grade	Prognosis	Cornea	Limbal Ischemia**

I	Good	Epithelial damage	None

II	Good	Haze, iris details visible	<1/3

III	Guarded	Stromal haze,

Iris details obscured	1/3 to ½

IV	Poor	Opaque cornea,

Iris and pupil obscured	>½

TABLE 3. Dua, King, and Joseph Classification of Severity of Ocular Surface Burns [30]

**Grade	Prognosis	Limbus Involvement	Conjunctival Involvement**

**(clock hours)	(%)**

I	Very good	0	0

II	Good	< or = 3	< or = 30

III	Good	>3–6	>30- 50

IV	Good-guarded	>6-9	>50-75

V	Guarded-poor	>9 to <12	>75 to <100

VI	Very poor	12 (total limbus)	100 (total conjunctiva)
